# Risk Assessment of Oropouche Virus Transmission by Mosquitoes in Europe

**DOI:** 10.1093/infdis/jiaf356

**Published:** 2025-07-08

**Authors:** Stephanie Jansen, Renke Lühken, Patrick Höller, Amrei Mack, Unchana Lange, Hanna Jöst, Norbert Becker, Anna Heitmann

**Affiliations:** Faculty of Mathematics, Informatics and Natural Sciences, University of Hamburg; Department of Arbovirology and Entomology, Bernhard Nocht Institute for Tropical Medicine, Hamburg; Department of Arbovirology and Entomology, Bernhard Nocht Institute for Tropical Medicine, Hamburg; Department of Arbovirology and Entomology, Bernhard Nocht Institute for Tropical Medicine, Hamburg; Department of Arbovirology and Entomology, Bernhard Nocht Institute for Tropical Medicine, Hamburg; Department of Arbovirology and Entomology, Bernhard Nocht Institute for Tropical Medicine, Hamburg; Department of Arbovirology and Entomology, Bernhard Nocht Institute for Tropical Medicine, Hamburg; Institute for Dipterology, Speyer; Centre for Organismal Studies, University of Heidelberg, Germany; Department of Arbovirology and Entomology, Bernhard Nocht Institute for Tropical Medicine, Hamburg

**Keywords:** Oropouche virus, vector competence, transmission risk

## Abstract

Oropouche virus (OROV) emerged as a significant health threat in Central and South America in 2024. *Culicoides* (Diptera: Ceratopogonidae) is considered the primary vector, while the role of mosquitoes in the transmission cycle is still unclear. This study evaluates the vector competence of 5 mosquito species common in Europe for OROV (old strain). While *Culex torrentium*, *Culex pipiens* biotype *pipiens*, *Aedes aegypti*, and *Aedes japonicus* showed no transmission, *Aedes albopictus* demonstrated low transmission at higher temperatures. Temperature-based risk analysis for areas with established *A albopictus* populations suggests that regions around the Mediterranean are at potential risk for OROV transmission.

## Teaser

The invasive mosquito species *Aedes albopictus* can transmit Oropouche virus at low levels but only at warm temperatures. This suggests a potential risk of Oropouche virus spread in Mediterranean regions with established populations of this vector.

The arbovirus Oropouche virus (OROV; *Orthobunyavirus oropoucheense*) has become a significant health concern in Central and South America [1, 2]. In February 2024, the Pan American Health Organization/World Health Organization issued an alert for an increasing number of human OROV infections. By the end of November 2024, >11 000 confirmed cases were reported, and for the first time, fatal outcomes and vertical transmission—likely leading to miscarriage and microcephaly—were described [1]. This prompted the Pan American Health Organization/World Health Organization to raise the alert level from medium to high, which emphasizes the urgency of preparedness, surveillance, and research to address critical knowledge gaps about OROV.

OROV belongs to the genus *Orthobunyavirus* (family Peribunyaviridae), the largest genus of known arboviruses. *Culicoides* (Diptera: Ceratopogonidae) is considered the primary vector of OROV, while the role of mosquitoes in the transmission cycle has not been clarified yet [2]. The few existing laboratory studies demonstrated low to no vector competence for *Culex quinquefasciatus*, *Culex pipiens*, *Anopheles quadrimaculatus*, *Aedes aegypti*, or *Aedes albopictus* [3–5]. However, there is a significant lack of knowledge, particularly regarding mosquitoes from Europe. At the same time, several travelers infected with OROV were identified in Europe importing OROV from Central and South America [1]. Therefore, we evaluated the vector competence for OROV for 5 mosquito species in Europe, including the native species *C pipiens* biotype *pipiens* and *Culex torrentium* as well as the invasive species *A albopictus*, *Aedes japonicus*, and *A aegypti*.

## METHODS

### Vector Competence Studies

Five species were investigated: 2 laboratory strains, *A albopictus* (from a population in Heidelberg, Germany, that has been in colony since 2016–2017) and *A aegypti* (as provided by Bayer and established several decades ago with origin unclear), as well as 3 species collected in the field in 2024 from the egg rafts of *C pipiens* biotype *pipiens* and *C torrentium* (Hamburg, Germany) and the eggs of *A japonicus* (Weinheim, Germany). A blood meal was offered via a feeder with a final concentration of 8.9 × 10^7^ plaque-forming units/mL of OROV strain TR 9760, which had been isolated from a febrile patient in 1955 and was currently produced in Vero cells (CCL-81) and titrated via the 50% tissue culture infectious dose (both obtained from ATCC); afterward, a salivation assay was performed as described by Heitmann et al [6]. Engorged females were incubated at 70% humidity at an oscillating temperature of 27 ± 5 °C, mimicking day-night variations (light:dark period of 12:12 hours). At 14 or 21 days postinfection (dpi), the following were determined: infection rate (IR; number of OROV-positive bodies per number of engorged females), transmission rate (TR; number of specimens with OROV-positive saliva per number of specimens with OROV-positive body), and transmission efficiency (TE; number of specimens with OROV-positive saliva per number of engorged specimens). RNA was quantified by quantitative reverse transcription polymerase chain reaction for the L segment as reported by Ciuoderis et al [7] but with VetMAX Xeno (Applied Biosystems, Thermo Fisher Scientific Corporation) as positive control.

### Risk Analysis

The potential risk map for OROV transmission in Europe was estimated by identifying areas presenting the temperature data used in the vector competence studies and the areas already colonized by *A albopictus*. Daily mean temperature data (European Reanalysis and Observations for Monitoring, E-OBS version 31.0e) were obtained from http://www.ecad.eu [8]. These E-OBS data, available on a 0.1° regular latitude-longitude grid, were extracted for a 5-year period from 2020 to 2024. For each grid cell, the number of days per year with the preceding 14 days having a mean daily temperature ≥24 °C or 21 days having a mean daily temperature ≥27 °C, respectively, were calculated. The annual values were then averaged over the 5-year period. The distribution data of *A albopictus* at the regional administrative level (NUTS3), as of July 2024, were obtained from the European Centre for Disease Prevention and Control [9]. All calculations and visualizations were conducted with the program R [10].

## RESULTS

### OROV Vector Competence Studies for Mosquitoes From Europe

No infection was observed for *A japonicus* (Table 1). Only 1 time point, 21 dpi, was tested for *A japonicus* due to the limited availability of specimens from the field. The 4 other tested species were susceptible for OROV infection. Positive specimens of *A aegypti*, *C pipiens* biotype *pipiens*, and *C torrentium* were exclusively detected at 14 dpi but not 21 dpi, with IRs of 6.5%, 14.3%, and 15.0%, respectively. In contrast, *A albopictus* showed an increasing IR from 6.7% at 14 dpi to 27.6% at 21 dpi at 27 °C. At 24 °C, the IR decreased from 6.4% at 14 dpi to 2.9% at 21 dpi. At 21°, positive specimens of *A albopictus* were also exclusively detected at 14 dpi, with an IR of 6.7%. At the lowest investigated temperature of 18 °C, no positive specimens were detected. Transmission of OROV was observed for *A albopictus* at 27 °C at 21 dpi, with a TR of 12.5% and a TE of 3.5%, and at 24 °C at 14 dpi, with a TR of 25% and a TE of 2.4%. Notably, with 9.5 (27 °C) and 8.4 (24 °C) RNA log_10_ copies per mosquito, the specimens with the positive saliva had the highest titers among all tested bodies.

**Table 1.** Infection and Transmission Rates of 5 Mosquito Species Experimentally Infected With OROV

Temperature, °C: Species	No.^a^	IR^b^	Body Titer Log_10_ RNA Copies / Mosquito Specimen, Mean (95% CI)	TR^c^	TE^d^
27 ± 5					
*Cx pipiens* biotype *pipiens*					
14 dpi	21	14.3	7.4 (6.9–7.9)	0.0	0.0
21 dpi	26	0.0	NA^e^	0.0	0.0
*Cx torrentium*					
14 dpi	20	15.0	7.1 (3.2–11.0)	0.0	0.0
21 dpi	16	0.0	NA	0.0	0.0
*A aegypti*					
14 dpi	31	6.5	4.6 (3.7–5.5)	0.0	0.0
21 dpi	29	0.0	NA	0.0	0.0
*A japonicus*					
21 dpi	30	0.0	NA	0.0	0.0
*A albopictus*					
14 dpi	31	3.2	8.0 (NA)	0.0	0.0
21 dpi	29	27.6	6.9 (5.5–8.3)	12.5	3.5
24 ± 5					
*A albopictus*					
14 dpi	41	6.4	7.1 (6.0–8.1)	25.0	2.4
21 dpi	35	2.9	7.0 (NA)	0.0	0.0
21 ± 5					
*A albopictus*					
14 dpi	30	6.7	5.3 (3.1–7.5)	0.0	0.0
21 dpi	28	0.0	NA	0.0	0.0
18 ± 5					
*A albopictus*					
14 dpi	30	0.0	NA	0.0	0.0
21 dpi	31	0.0	NA	0.0	0.0

Abbreviations: dpi, days postinfection; IR, infection rate; NA, not applicable; OROV, Oropouche virus; TE, transmission efficiency; TR, transmission rate.

^a^No. of engorged specimens

^b^Infection rate: No. of OROV-positive bodies per number of engorged females

^c^Transmission rate: No. of specimens with OROV-positive saliva per number of specimens with OROV-positive bodies.

^d^Transmission efficiency: No. of specimens with OROV-positive saliva per engorged specimens.

^e^Not applicable for the mean if there were no positive specimens or for the 95% CI if there was only 1 positive body.

### Risk Analysis

**Figure 1.**
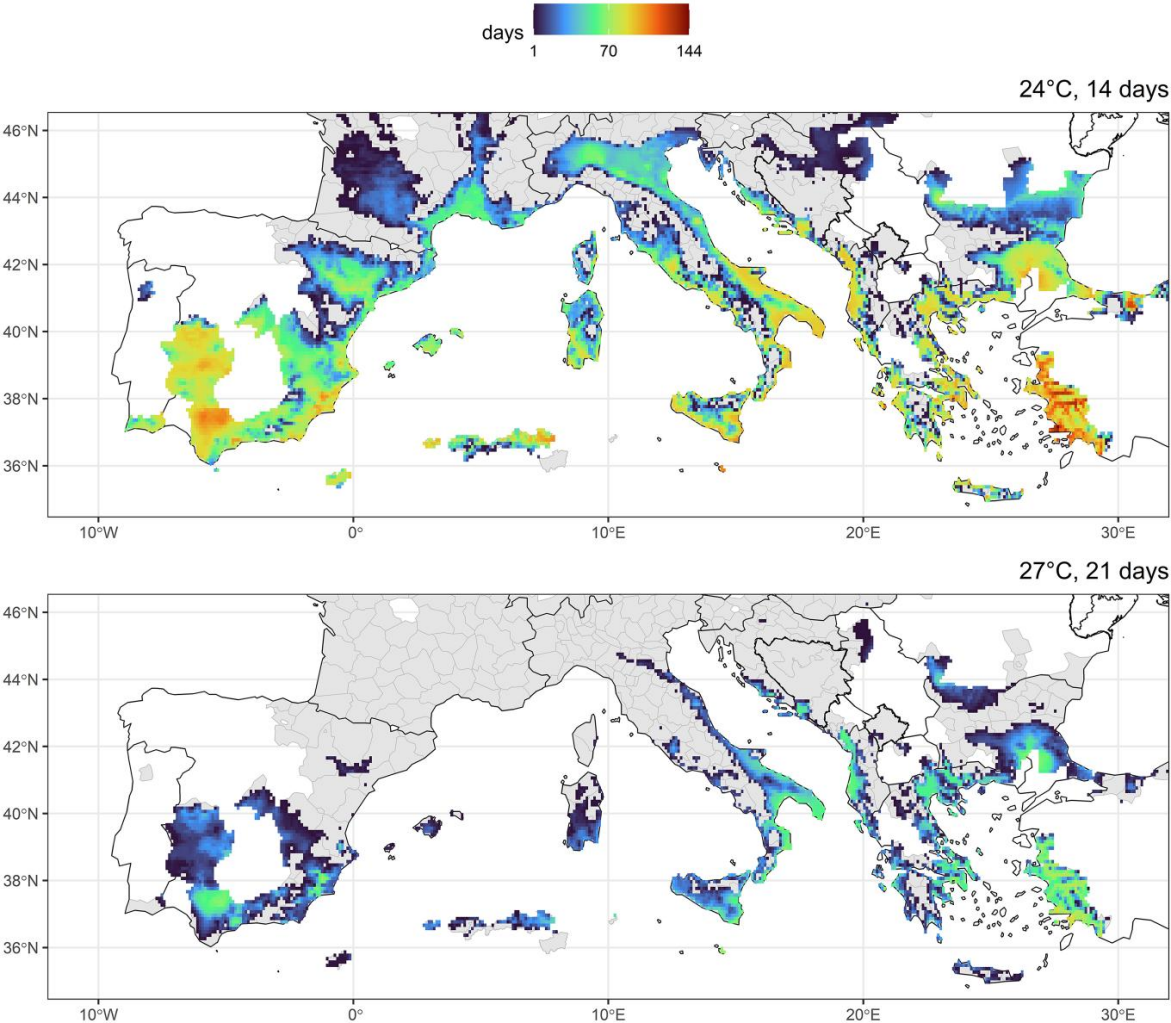
Risk assessment of Oropouche virus transmission in Europe. Average number of days per year with the preceding 14 days having a mean daily temperature ≥24 °C or 21 days having a mean daily temperature ≥27 °C for the 2020–2024 period for the current distribution of *Aedes albopictus* in Europe. Areas with established populations but without reaching this temperature condition are indicated in gray.

The average number of days per raster cell (2020–2024) with the preceding 21 days having a mean daily temperature ≥27 °C was observed only for restricted areas within the current distribution of *A albopictus* in Europe (Figure 1). In contrast, 14 days with a mean daily temperature ≥24 °C were observed for a much wider area. Regions directly bordering the Mediterranean Sea were the most suitable risk areas, with the longest periods observed for Spain, Southern Italy, Greece, and Turkey.

## DISCUSSION

Our study shows that 2 common European *Culex* species, *C pipiens* biotype *pipiens* and *C torrentium*, and the invasive *A aegypti* are susceptible to OROV infection on a low level. No transmission was detected for the tested *Culex* species and *A aegypti*. This is consistent with results for *C tarsalis* and *C pipiens* biotype *molestus/C pipiens* [3–5]. Interestingly, Payne et al showed transmission of OROV by *C pipiens* but only for the current outbreak OROV strain, not the old OROV strain, which was used in our study [5]. As the number of investigated specimens is low, further studies are needed to interpret these results. Studies on *C quinquefasciatus* are more variable, ranging from no infection to low transmission rates after an artificial blood meal [3, 5]. In line with previous studies, the invasive *A aegypti* and *A japonicus* showed no transmission of OROV [3–5].

Transmission of OROV was observed only for *A albopictus*. Interestingly, transmission was exclusively observed 21 dpi at 27 °C and 14 dpi at 24 °C. This shows that transmission by *A albopictus* is possible at both temperatures but rarely. Due to the small number of analyzed individuals (n = 30), an exact temporal prediction is not possible. Further experiments on the extrinsic incubation period should be performed. Our results are in contrast to the literature, where no or low OROV infection but no transmission by *A albopictus* was detected after an artificial blood meal [3, 5]. Such differences in vector competence of different *A albopictus* populations are also shown for Zika virus and chikungunya virus [11]. Similarly, different virus strains can result in a different transmission potential of mosquito populations [11]. Additionally, the way of infection (live animal, artificial blood meal, intrathoracic injection) has an important influence on the infection with OROV, whereas the artificial blood meal may be less effective as other methods, as reviewed by Gallichotte et al [3]. The current OROV outbreak lineage is identified as an intraspecies reassortant of 2 OROV lineages [12]. It has been discussed whether the new virus variant has possibly resulted in a worsening of the clinical presentation of human OROV infections and allowed the better transmission by mosquitoes [3, 5]. However, first studies comparing an old OROV strain with the new variant do not show implications for an increased competence of mosquitoes [5]. Further studies are necessary to clarify if the transmission capability of vectors has changed and if the spectrum of potential vectors has expanded.

The vector competence of an *A albopictus* population from Central Europe for OROV highlights the need to assess the risk of autochthonous OROV transmission in Europe, although the unclear trend across temperatures and days postinfection indicates that the species is probably not an extremely good vector for OROV. However, the widespread establishment of *A albopictus* increases the risk of transmission of tropical viruses, as observed for local outbreaks of dengue, Zika, and chikungunya virus, all linked to infected travelers and established populations of *A albopictus* [13]. Long-term local establishment of these viruses has not been observed so far and is unpredictable for OROV—that is, although several species are considered amplifying and reservoir hosts for OROV, their exact epidemiologic role is not well understood [14]. During the huge OROV outbreak in Central and South America in 2024, travelers infected with OROV were also detected in various European countries where established populations of *A albopictus* exist [1]. A joint analysis of the current vector distribution and temperature data indicates that regions around the Mediterranean Sea are at higher risk of autochthonous OROV transmission, with hotspots in Spain, Italy, and Turkey having longer periods of average daily temperatures >24 °C.

## Notes

***Acknowledgments.*** We thank Anucha Ponyiam for his work in the mosquito-breeding facility and Corinna Thomé-Bolduan for her great support with the cultivation of OROV.

***Author contributions.*** S. J., R. L., and A. H. conceived and designed the experiments. S. J., P. H., A. M., U. L., and A. H. performed the laboratory experiments. R. L., H. J., and N. B. performed fieldwork. R. L., P. H., and A. H. analyzed the data. R. L. and A. H. ensured the funding and contributed reagents, materials, and analysis tools. S. J., R. L., and A. H. critically discussed the results and wrote the original draft. P. H., A. M., U. L., H. J., and N. B. critically revised and edited the manuscript. All authors read and approved the final manuscript for publication.

***Financial support.*** This work was supported by the Federal Ministry of Education and Research (BMBF, Germany) (grant 01Kl2022 to R. L.); and the Federal Ministry of Food and Agriculture (BMEL, Germany) through the Federal Office for Agriculture and Food (BLE, Germany) (financial support to P. H. and U. L. from grant FKZ 2819107A22 to R. L. and A. H.).
